# Signaling Pathways That Regulate the Crustacean Molting Gland

**DOI:** 10.3389/fendo.2021.674711

**Published:** 2021-06-21

**Authors:** Donald L. Mykles

**Affiliations:** ^1^ Department of Biology, Colorado State University, Fort Collins, CO, United States; ^2^ University of California-Davis Bodega Marine Laboratory, Bodega Bay, CA, United States

**Keywords:** Y-organ, molting (control of), mTOR - mammalian Target of Rapamycin, ecdysteroid, neuropeptide, insulin, growth factor, G protein coupled receptor (GPCR)

## Abstract

A pair of Y-organs (YOs) are the molting glands of decapod crustaceans. They synthesize and secrete steroid molting hormones (ecdysteroids) and their activity is controlled by external and internal signals. The YO transitions through four physiological states over the molt cycle, which are mediated by molt-inhibiting hormone (MIH; basal state), mechanistic Target of Rapamycin Complex 1 (mTORC1; activated state), Transforming Growth Factor-β (TGFβ)/Activin (committed state), and ecdysteroid (repressed state) signaling pathways. MIH, produced in the eyestalk X-organ/sinus gland complex, inhibits the synthesis of ecdysteroids. A model for MIH signaling is organized into a cAMP/Ca^2+^-dependent triggering phase and a nitric oxide/cGMP-dependent summation phase, which maintains the YO in the basal state during intermolt. A reduction in MIH release triggers YO activation, which requires mTORC1-dependent protein synthesis, followed by mTORC1-dependent gene expression. TGFβ/Activin signaling is required for YO commitment in mid-premolt. The YO transcriptome has 878 unique contigs assigned to 23 KEGG signaling pathways, 478 of which are differentially expressed over the molt cycle. Ninety-nine contigs encode G protein-coupled receptors (GPCRs), 65 of which bind a variety of neuropeptides and biogenic amines. Among these are putative receptors for MIH/crustacean hyperglycemic hormone neuropeptides, corazonin, relaxin, serotonin, octopamine, dopamine, allatostatins, Bursicon, ecdysis-triggering hormone (ETH), CCHamide, FMRFamide, and proctolin. Contigs encoding receptor tyrosine kinase insulin-like receptor, epidermal growth factor (EGF) receptor, and fibroblast growth factor (FGF) receptor and ligands EGF and FGF suggest that the YO is positively regulated by insulin-like peptides and growth factors. Future research should focus on the interactions of signaling pathways that integrate physiological status with environmental cues for molt control.

## Introduction

The progression of decapod crustaceans through the molt cycle depends on ecdysteroids synthesized by the Y-organ [YO; reviewed in (1)]. The molt cycle is unidirectional, progressing from the intermolt stage through premolt, ecdysis, and postmolt stages to the next intermolt stage [reviewed in (2, 3)]. Molting encompasses the preparatory processes during the premolt stage, culminating with the actual shedding of the exoskeleton (ecdysis), followed by restorative processes during the postmolt stage. Rising titers of ecdysteroids in the hemolymph initiate and coordinate premolt processes, such as synthesis of the new exoskeleton, degradation and resorption of the old exoskeleton, claw muscle atrophy, and limb regenerate growth (2, 4, 5). A precipitous drop in hemolymph ecdysteroids at the end of premolt triggers ecdysis (1). The low ecdysteroid titer during postmolt allows claw muscle growth and completion of exoskeleton synthesis and its calcification. Intermolt can last from weeks to years in adult decapods.

**Figure 1:**
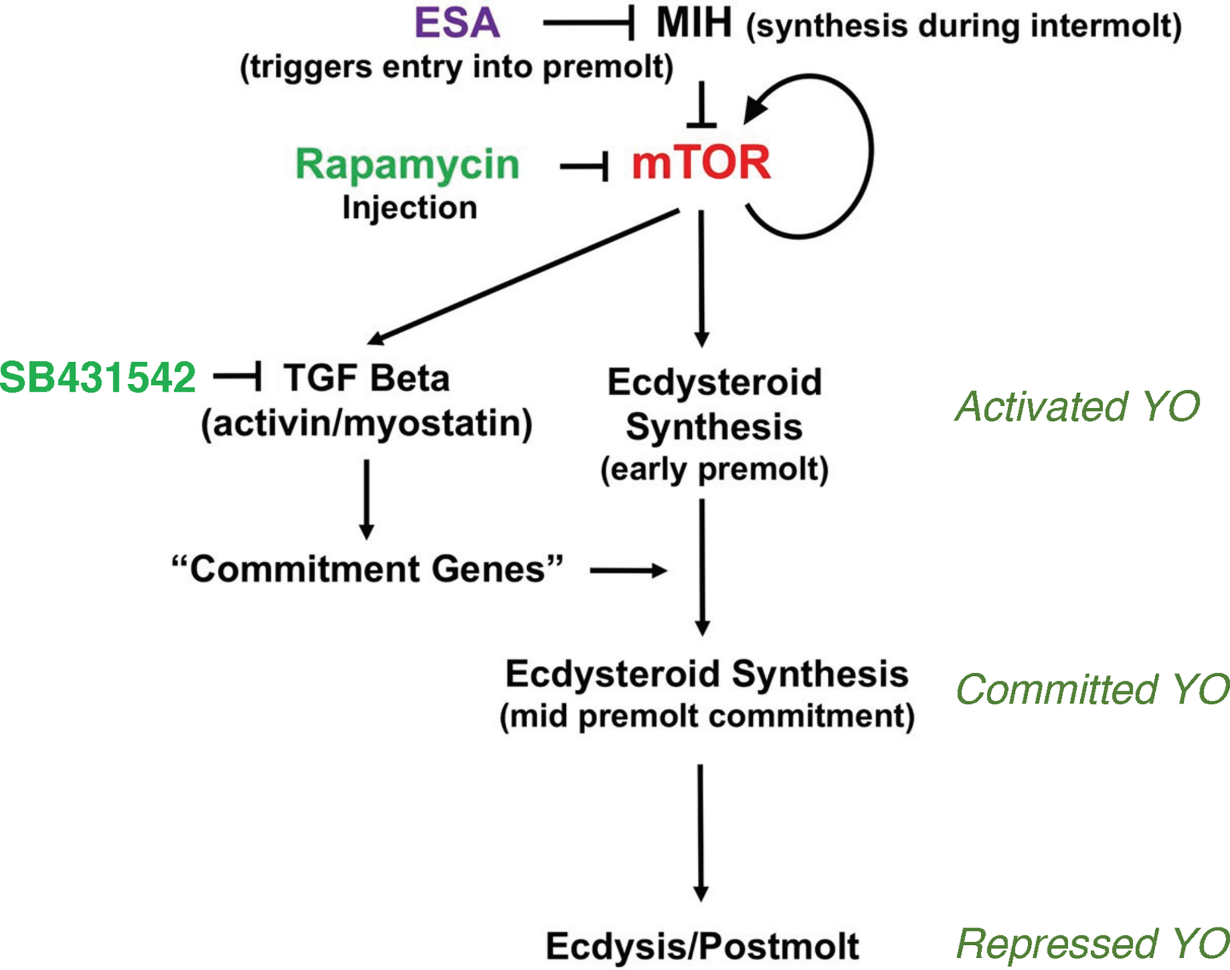
Organization of the signaling pathways mediating YO phenotype transitions over the molt cycle. Cyclic nucleotide-mediated MIH signaling maintains the YO in the basal state by inhibiting mTOR signaling. Reduction in MIH, such as by eyestalk ablation (ESA), stimulates mTOR activity, which is inhibited by rapamycin. mTOR stimulates ecdysteroid synthesis and up-regulates mTOR and TGFβ/Activin signaling genes, and down-regulates MIH signaling genes. Activin/myostatin signaling, which is inhibited by SB431542, up-regulates mTOR signaling genes and controls expression of commitment genes that determine the committed phenotype. High ecdysteroid titers in late premolt may trigger the repressed phenotype in postmolt. From (2).

Molt stage transitions are determined by phenotypic changes in the activity and properties of the YO. In the intermolt stage (stage C_4_), inhibitory neuropeptides produced in the X-organ/sinus gland complex, such as molt-inhibiting hormone (MIH) and crustacean hyperglycemic hormone (CHH), maintain the YO in the basal state (**Figure 1**). A proposed model for MIH signaling couples a cAMP/Ca^2+^-dependent triggering phase with a NO/cGMP-dependent summation phase [reviewed in (2)]. The prolonged activation of a calmodulin-dependent NO synthase and NO-dependent guanylyl cyclase (GC-I) represses ecdysteroidogenesis between MIH pulses (2, 6–9). The decision to molt, or enter premolt, is determined by integration of environmental and physiological cues by the central nervous system that are not completely understood (10). A decrease in MIH release by the X-organ/sinus gland complex, which can be experimentally induced by eyestalk ablation (ESA), triggers YO activation and entry into early premolt (stage D_0_) (2, 3, 11). Multiple limb autotomy (MLA) also induces molting, as limb regenerates only become functional appendages when extended at ecdysis (4, 12, 13). It is hypothesized that MLA-induced molting is mediated by a stimulatory factor, designated limb autotomy factor – anecdysis (LAF_an_), produced by the developing limb buds (3). YO activation requires mechanistic Target of Rapamycin Complex 1 (mTORC1) activity, as rapamycin inhibits YO ecdysteroidogenesis *in vitro* and prevents YO activation *in vivo* (14, 15). mTORC1-dependent protein synthesis drives the initial increase in ecdysteroid synthesis by the YO. The activated YO remains sensitive to MIH, CHH, and other factors, giving the animal the flexibility to suspend or delay molting when conditions turn unfavorable (2).

A critical decision point occurs at the end of early premolt, when the animal becomes committed to molt. The transition of the YO from the activated to the committed state is mediated by transforming growth factor beta (TGFβ)/Activin signaling, as SB431542, an inhibitor of Activin receptor signal transduction, prevents progression of animals from early premolt to mid-premolt (stage D_1_; **Figure 1**) (15). mTORC1 activity affects the mRNA levels of thousands of genes, including those in the mTORC1 and TGFβ/Activin signaling pathways (**Figure 1**) (16). An invertebrate Myostatin (Mstn)-like factor, first described in scallop and crustacean muscles (17–19), appears to be the ligand for the Activin receptor. It is highly expressed in the YO and its mRNA levels are highest in the activated YO (15, 20, 21). The committed YO increases ecdysteroid synthesis, resulting in increasing ecdysteroid titers in the hemolymph during mid-premolt and reaching a peak in ecdysteroid titer at the end of late premolt (1). The committed YO also becomes insensitive to MIH and CHH (2). Limb bud autotomy, which suspends molting processes in early premolt, is no longer effective in mid- and late premolt animals (2, 12, 13).

The signaling mechanisms controlling the transition of the committed YO to the repressed YO at the end of late premolt and the transition of the repressed YO to the basal YO at the end of postmolt are not well understood. It is hypothesized that the large peak in hemolymph ecdysteroid titer triggers the transition to the repressed phenotype (**Figure 1**), as the YO expresses the ecdysteroid receptor (EcR/RXR) and ecdysteroid-responsive genes (2). The repressed YO has low ecdysteroid synthetic activity, which results in low hemolymph ecdysteroid titers during postmolt (1). Most of the 478 differentially-expressed genes assigned to signal transduction pathways are down-regulated to their lowest levels during the postmolt stage (21). Among these are critical components of the MIH, mTORC1, and TGFβ/Activin signaling pathways (21). These data suggest that the YO is not inhibited by MIH during postmolt and that repression of the YO involves transcriptional regulation that prevents premature reactivation of the YO until exoskeleton synthesis and calcification are completed (2). The model assumes that normal MIH control is not restored until the YO returns to the basal state in intermolt.

Transcriptomics and proteomics have revolutionized crustacean physiology (22, 23). These approaches have shown that the YO undergoes molt stage-specific changes in phenotype that differ quantitatively and qualitatively in mRNA and protein levels (2, 16, 21, 24). mTORC1 activity plays a critical role in controlling ecdysteroid synthesis at the transcriptional and translational levels (2, 14, 16). Transcriptomics and proteomics can also be tools for discovery. Analysis of the MLA *Gecarcinus lateralis* YO transcriptome identified 878 unique contigs assigned to 23 KEGG signaling pathways, including those for MIH/CHH, mTOR, and TGFβ/Activin [**Table 1**; (21)]. The YO also expresses MAP kinase, AMP kinase, ErbB, Hedgehog, HIF-1, Jak-STAT, Hippo, NF-kappa B, Notch, TNF, and Wnt signaling pathway genes among others, raising the possibility that ecdysteroidogenesis is regulated by a great many factors (20, 21). Proteomic analysis has revealed that anti-radical oxygen species, cytoskeletal, vesicular secretion, immune response, protein homeostasis proteins contribute to *G. lateralis* YO function (24). This review presents the current knowledge of the signaling pathways that control ecdysteroid synthesis by the YO and identifies areas for future research. It includes relevant research on signaling mechanisms that control the insect prothoracic gland.

**Table 1:** Number of total and differentially expressed annotated contigs in the *G. lateralis* YO transciptome assigned to KEGG signal transduction pathways. From (21).

Signaling Pathway	Pathway ID	Number of annotated contigs	Number of DE contigs (percentage)
**AMPK signaling pathway**	k04152	86	53 (62%)
**Calcium signaling pathway**	k04020	92	49 (53%)
**cAMP signaling pathway**	k04024	104	65 (63%)
**cGMP-PKG signaling pathway**	k04022	94	51 (54%)
**ErbB signaling pathway**	k04012	48	24 (50%)
**FoxO signaling pathway**	k04068	79	46 (58%)
**Hedgehog signaling pathway**	k04340	36	21 (58%)
**HIF-1 signaling pathway**	k04066	50	26 (52%)
**Hippo signaling pathway**	k04390	95	52 (55%)
**Jak-STAT signaling pathway**	k04630	26	12 (46%)
**MAPK signaling pathway**	k04010	106	66 (62%)
**mTOR signaling pathway**	k04150	92	54 (59%)
**NF-kappa B signaling pathway**	k04064	33	16 (48%)
**Notch signaling pathway**	k04330	28	17 (61%)
**Phosphatidylinositol signaling system**	k04070	86	47 (55%)
**Phospholipase D signaling pathway**	k04072	83	50 (60%)
**PI3K-Akt signaling pathway**	k04151	128	69 (54%)
**Rap1 signaling pathway**	k04015	115	63 (55%)
**Ras signaling pathway**	k04014	105	57 (54%)
**Sphingolipid signaling pathway**	k04071	98	60 (61%)
**TGF-beta signaling pathway**	k04350	32	18 (56%)
**TNF signaling pathway**	k04668	40	23 (58%)
**Wnt signaling pathway**	k04310	79	51 (65%)

## G Protein-Coupled Receptor-Mediated Signaling

Transcriptomic analysis has revealed that a large number of G protein-coupled receptors (GPCRs) are expressed in decapod crustacean tissues. GPCRs are characterized by seven transmembrane domains, an external N-terminal domain, and a C-terminal cytosolic domain (25). These are divided among three large classes: rhodopsin-like (Class A), which represents the largest number of GPCRs; secretin-like (Class B); and metabotropic glutamate (Class C) (25–44).

All three GPCR classes are expressed in the YO, but represent a subset of those cataloged in decapod tissues (25, 29). In green shore crab *Carcinus maenas*, 62 contigs encoding GPCRs were identified in the central nervous system (29). The YO expresses 37 GPCRs annotated to 17 ligand clusters (**Table 2**). Thirty-two contigs are Class A and 5 contigs are in Class B; no Class C contigs were identified in the *C. maenas* YO (**Table 2**) (29). Eleven GPCRs are enriched in the YO compared to the epidermis; these were identified as gonadotropin-releasing hormone receptor, tachykinin-like R86C, relaxin R1, two rhodopsin G0-coupled receptors, two methuselah-like R1, dopamine D2-like receptor, opsin UV-sensitive receptor, serotonin R4, and GPCR161 (29). In addition, seven GPCRs are differentially expressed over the molt cycle; these include short neuropeptide F, Bursicon R2, CHHa R1, relaxin R3, ITPR-like, Moody-like, and Ast-B/MIP-R1 (29). Deep high throughput RNA sequencing and *de novo* assembly of the intermolt *G. lateralis* YO identified 99 putative GPCRs, 65 of which were annotated to 32 ligand clusters (**Table 2**) (25). The ligands are mostly neuropeptides, but also include biogenic amines, such as dopamine, octopamine, and serotonin (**Table 2**). These data suggest that the YO can potentially respond to a wide variety of ligands. The possible roles of some of these GPCRs are discussed in the sections below.

**Table 2:** Classification and number of contigs encoding G protein-coupled receptors in the *Gecarcinus lateralis* and *Carcinus maenas* Y-organ transcriptomes.

Predicted receptor	*G. lateralis*	*C. maenas*
**Class A (Rhodopsin-like)**		
Adenosine	1	0
Allatostatins	3	4
CCAP	1	3
CCHamide	2	3
CHH/ITP	3	2
Corazonin	2	2
Dopamine	0	1
ETH	1	2
FMRFamide	2	1
HPR1	4	0
Leucine-rich repeats family		
Type A (GPA2/GPB5)	2	3
Type B (Lgr2; Bursicon)	1	3
Type C1 (Lgr3; Dilp8)	1	3
Type C2 (GRL101-like)	1	0
Moody	1	2
Myosuppressin	0	1
Octopamine	1	0
Peropsin	1	0
Proctolin	1	2
Prostaglandin	4	0
Serotonin (5-HT)	2	0
sNPF	1	1
Tre-1 (formerly TIE)	1	0
TRH	1	0
**Class B (Secretin-like)**		
DH31	1	4
DH41	1	0
Latrophilin	3	0
Lipoprotein	4	0
Methuselah	12	0
Parathyroid	1	0
PDF	3	1
**Class C (Metabotropic glutamate)**		
Boss	1	0
Mangetout	1	0
Metabotropic glutamate	1	0

CCAP, crustacean cardioactive peptide; CHH, crustacean hyperglycemic hormone; DH, diuretic hormone; ETH, ecdysis triggering hormone; HPR1, protein receptor in hepatopancreas 1; 5-HT, 5-hydroxytryptamine; ITP, ion transport peptide; PDF, pigment dispersing factor; sNPF, short neuropeptide F; Tre-1, trapped in endoderm-1; TRH, thyrotropin-releasing hormone. Data from (25, 29, 45).

Surprisingly, the YO expresses a variety of peptide hormones. In *C. maenas*, contigs encoding 19 full-length peptides were identified (29). The six peptides that are expressed at the highest levels are Neuroparsin-1, -3, and -4; CHH-1, inotocin/vasopressin, and Eclosion Hormone-2 (29). Further research should determine if the transcripts are translated into peptides and the peptides are secreted into the hemolymph. If so, it would provide compelling evidence that the YO has endocrine functions beyond that of ecdysteroid production.

### Putative G Protein-Couple Receptors for CHH Family Neuropeptides

Peptides in the CHH family are divided into two types that differ in the amino acid sequences of the precursor proteins. Members of this family, which includes insect ion transport peptide (ITP), have a 66-amino acid “CHH family motif” in the mature peptide with six conserved cysteines that form three intramolecular disulfide bridges to stabilize the structure of the native protein (46–50). Type I peptides, which include CHH and ITP, are characterized by a signal peptide sequence followed by CHH/ITP precursor-related peptide (CPRP) and mature peptide sequences. Type II peptides, which include MIH, gonad-inhibiting hormone (GIH), and mandibular organ-inhibiting hormone (MOIH), lack a precursor-related sequence and have a glycine inserted between residues #11 and #12 and an invariant valine at position #20 in the mature peptide (46). Recently, a comprehensive phylogenetic analysis of crustacean ITPs proposed that ITPs be assigned to a third group (Type III) distinct from Type I CHHs (48, 50). The solution structures of MIH and CHH are similar, except that a short alpha-1 helix at positions #10 through #13 in MIH is lacking in CHH (49, 51). It is thought that the Gly12 contributes to the formation of the alpha-1 helix in Type II peptides (51). The surface structures of the N- and C-terminal regions confer specificity of binding to distinct receptors in the YO membrane (47, 49, 51–54).

The identification and characterization of the MIH receptor has remained elusive for more than three decades (46, 55). It is hypothesized that the receptors for the CHH family are GPCRs, given the similar native structures of Type I and Type II peptides (49, 51) and signal transduction mediated by cyclic nucleotide second messengers (56). In insects, studies of silk moth GPCRs (*Bombyx* neuropeptide G-protein coupled receptors, or BNGRs) identified BNGR-A2 and -A34 as ITP receptors, and BNGR-A24 is an ITP-like receptor (57). Based on this discovery, two full-length contigs (Pc-GPCRA52 and A53) and one partial contig (Pc-GPCRA63) from the transcriptome of adult *Procambarus clarkii* were identified as putative CHH-like receptors (CHHRs) (58). Subsequently, CHHR orthologs from more than ten other decapod crustacean species have been identified (25, 27, 29).

**Figure 2:**
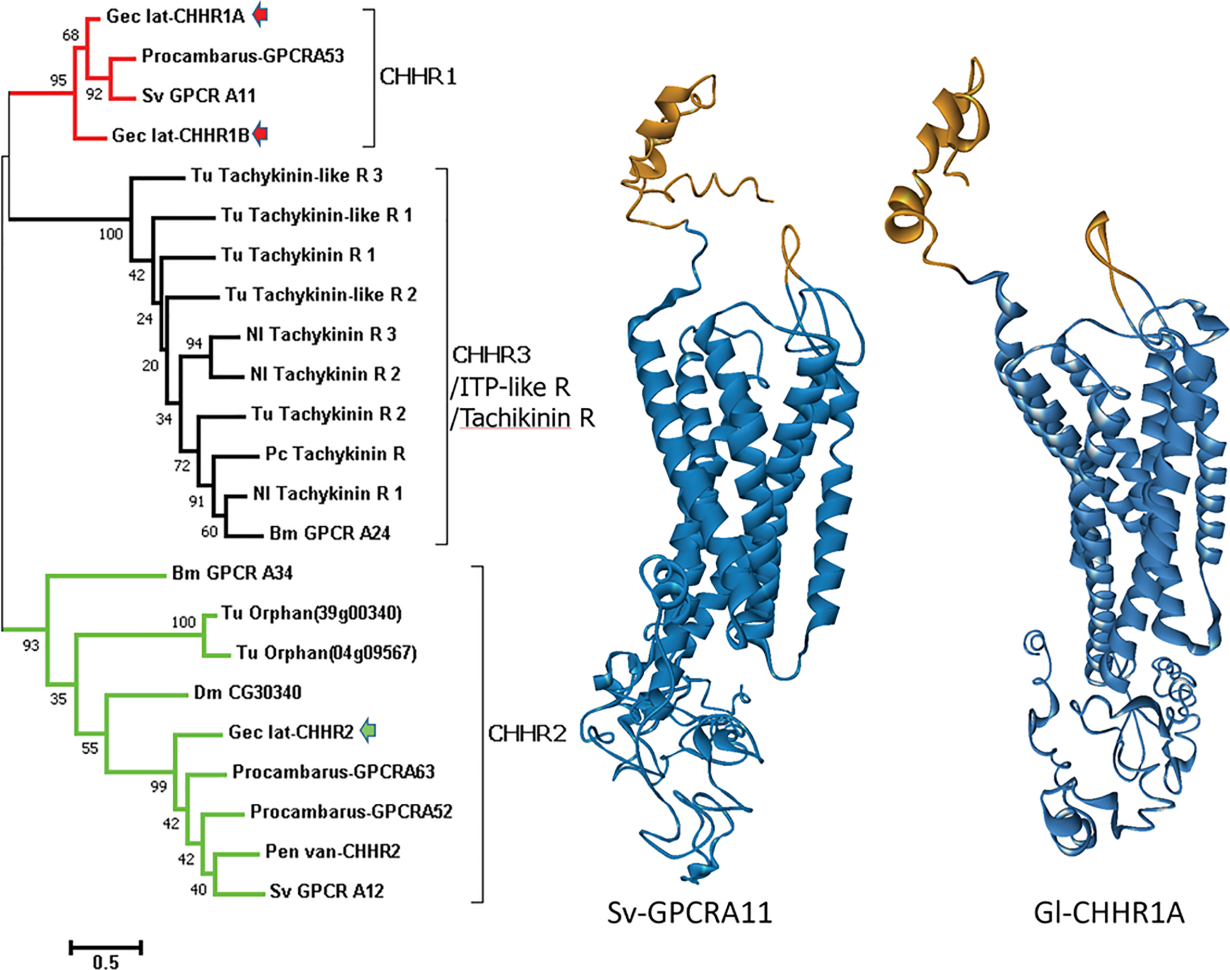
Comparison of decapod putative CHH and insect and mite ITP-like/tachykinin GPCRs. Left: A phylogram (left) of arthropod neuropeptide GPCRs showing three clusters designated CHHR1 (red), CHHR2 (green), and CHHR3/ITP-like R/tachykinin R (black). *G. lateralis* (Gec_lat) CHHR1A and 1B, originally designated Gl-GPCR-A9 and -A10, respectively (25), are within the CHHR1 cluster (red arrows) and CHHR2, originally designated Gl-GPCR-A12 (25), is within the CHHR2 cluster (green arrow). Species: Bm, *Bombyx mori*; Dm, *Drosophila melanogaster*; Nl, *Nilaparvata lugens*; Pen van, *Penaeus* (*Litopenaeus*) *vannamei*; Procambarus, *P. clarkii*; Sv, *S. verreauxi*; and Tu, *Tetranychus urticae* (red spider mite). Right: Structural models comparing the putative CHHR1 proteins from two decapod species predicted by GPCR-I-TASSER (27): *S. verreauxi* (Sv-GPCRA11) and *G. lateralis* Gl-CHHR1A. Gold indicates the N-terminal neuropeptide binding cleft in the extracellular domain. Blue indicates the transmembrane domain containing the 7 α-helices characteristic of GPCRs and the C-terminal intracellular domain. Figure provided by Dr. Tomer Ventura.

Three putative CHHRs, designated Gl-GPCR-A9, -A10, and -A12, are expressed in the *G. lateralis* YO transcriptome (25). Two CHH/ITP-like receptors were identified in the *C. maenas* YO transcriptome (29). Further phylogenetic analysis showed that the arthropod CHH/ITP GPCRs formed three clusters, designated CHHR1, CHHR2, and CHHR3/ITP-like R/tachykinin R (**Figure 2**). As Gl-GPCR-A9 and -A10 were grouped in the CHHR1 cluster, they are renamed Gl-CHHR-1A and -1B, respectively; Gl-GPCR-A12 is renamed Gl-CHHR-2 (**Figure 2**). Modeling the 3-dimensional structures of the *G. lateralis* CHHRs gave a highly conserved outcome of a predicted cleft at the N-terminus, suggesting a common role in binding CHH family hormones (**Figure 2**). End-point PCR showed that neither *Gl-CHHR-1A* or *-2* are exclusively expressed in the YO, as would be expected for the MIH receptor; *Gl-CHHR-1B* was not examined (25). Interestingly, the YO is the only tissue to express both CHHRs. Eyestalk ganglia, thoracic ganglion, gill, heart, and midgut only express *Gl-CHHR-1A*; testis, hindgut, and hepatopancreas only express *Gl-CHHR-2*; and claw muscle does not express either CHHR (25). The three *G. lateralis* CHHRs are differentially expressed in the YO over the molt cycle, which suggests altered sensitivities to CHH neuropeptides associated with YO phenotypic changes. *Gl-CHHR-1A* has higher expression in intermolt and decreases during premolt stages; *Gl-CHHR-1B* is expressed during intermolt with higher expression during premolt stages; and *Gl-CHHR-2* is expressed at high levels at late premolt (25). None of the *Gl-CHHRs* are expressed during postmolt (25). One of the CHH/ITP-like receptors in the *C. maenas* YO is differentially expressed over the molt cycle, with highest expression in late premolt (29). Future research must use a functional assay to establish which, if any, of the GPCR candidates is the MIH receptor.

### Corazonin Receptor

Corazonin (CRZ) is a conserved 11- amino acid neuropeptide with an amidated C-terminus and pGlu at the N-terminus. The sequence (pQTFQYSRGWTNa) is highly conserved among insect and crustacean species, although variants with single amino acid substitutions occur in some insects (59, 60). In *Drosophila melanogaster*, CRZ neurons modulate prothoracicotropic hormone (PTTH) action on basal ecdysteroidogenesis by the prothoracic gland (PG), thus controlling larval growth without affecting metamorphosis (61). *C. maenas* corazonin receptor (*Cm-CRZR*) is primarily expressed in the YO, suggesting that CRZ plays a role in regulating ecdysteroidogenesis (45). However, CRZ peptide (50 nM), which is produced in the eyestalk ganglia and other areas of the central nervous system, has only a small stimulatory effect on YO ecdysteroidogenesis in postmolt (stages A-B) *C. maenas* (45).

### Leucine-Rich Repeat Receptor and Insulin-Like Peptides

Tissue loss or injury delays molting, allowing time for regeneration or regrowth of tissues or organs prior to the next ecdysis. Molting delay by limb bud autotomy (LBA) in crustaceans and injury to imaginal discs in insects allows time for tissue regeneration, while growth of remaining or undamaged tissues slows or stops (12, 13, 62–66). In crustaceans, LBA during early premolt (stage D_0_) suspends premolt two to three weeks by lowering hemolymph ecdysteroid titers, so that animals can regain a full set of functional claws and legs at ecdysis (13, 67). In *G. lateralis*, secondary LBs produce a factor, designated Limb Autotomy Factor – proedysis (LAF_pro_), that lowers hemolymph ecdysteroid (3, 12, 13). In insects, regenerating imaginal discs produce a factor, identified as *Drosophila* insulin-like peptide 8 (Dilp8) in *D. melanogaster*, that delays metamorphosis by lowering ecdysteroid synthesis by the prothoracic gland (PG) (64, 68–70). Dilp8 also delays molting by activating Lgr3 neurons in the brain, which inhibit PTTH synthesis in PTTH neurons (71–74).

**Figure 3:**
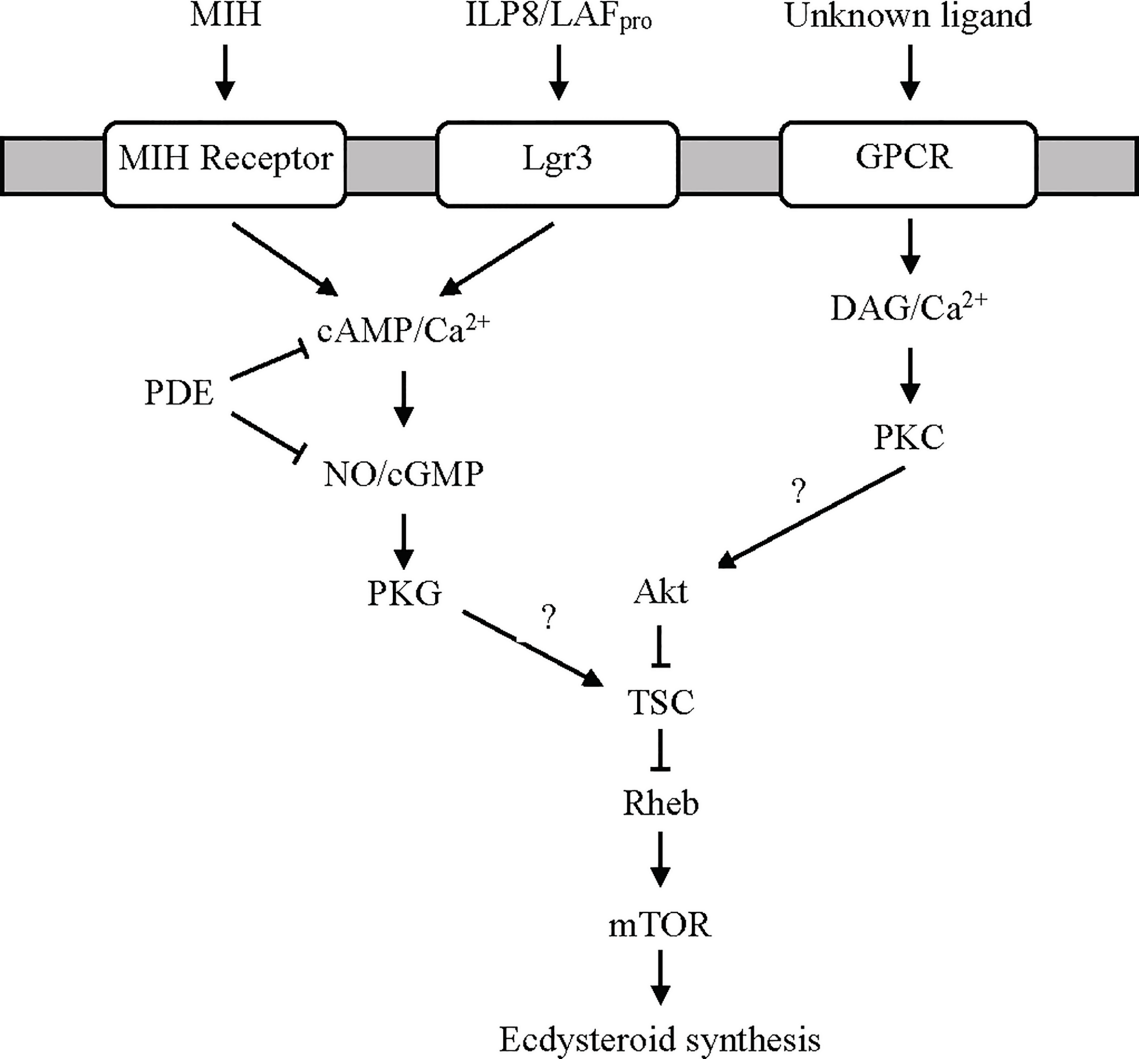
Proposed G protein-coupled receptor-mediated signaling pathways regulating ecdysteroidogenesis in the YO. MIH and limb autotomy factor - proecdysis (LAF_pro_) activate cyclic nucleotide/NO-dependent signaling *via* distinct receptors. It is hypothesized that LAF_pro_, secreted by secondary limb regenerates during early premolt, is an insulin-like peptide similar to dILP8 in *Drosophila* that binds to Lgr3. Cyclic phosphodiesterase (PDE) activity inhibits MIH and LAF_pro_ signaling by hydrolyzing cAMP and cGMP to AMP and GMP, respectively. An unknown ligand, possibly serotonin or other biogenic amines (see Ca2+/Diacylglycerol/Protein Kinase C Signaling Section), binds a GPCR to activate the Ca^2+^/diacylglycerol (DAG)/protein kinase C (PKC) pathway. Both pathways converge on mTOR signaling, possibly by phosphorylation of tuberous sclerosis complex (TSC) by protein kinase G (PKG) to inhibit ecdysteroid synthesis or by phosphorylation of Akt by PKC to stimulate ecdysteroid synthesis (2, 12, 50, 84).

The LAF_pro_ signaling pathway has not been fully characterized, but parallels with the action of Dilp8 on the insect PG suggest a common mechanism. Dilp8 is one of eight insulin-like peptides (ILPs) in *D. melanogaster* (74–78). The ILP superfamily consists of insulin, insulin-like growth factors (IGFs), and relaxin-like peptides (38, 71). Dilp 1 to 6 are in the insulin/IGF clade and bind to receptor tyrosine kinase receptors; Dilp 7 and 8 are in the relaxin-like clade and bind to leucine-rich repeat GPCRs (71, 74, 75). LAF_pro_ is a peptide that is distinct from MIH. MIH is resistant to boiling in deionized water or weak acids (see (13) for references). LAF_pro_ is stable when boiled 15 min in deionized water, but is inactivated by boiling in 0.1 M acetic acid (pH 2.9) and by proteinase K digestion (13). Dilp8 action is mediated through relaxin receptor Lgr3 and activation of NOS (73, 74, 79). NO donors inhibit ecdysteroidogenesis in both insect and crustacean molting glands (80–82). Dilp8 binding to Lgr3 stimulates production of cAMP in *Drosophila* cells (71). Up-regulation of *Dilp8* delays expression of Halloween genes *disembodied* (*Dib*) and *phantom* (*Phm*) in the PG (68, 69). Moreover, targeted tissue damage or NOS overexpression in the PG lowers the expression of Halloween genes *spookier* (*Spok*) and *Dib* (79). These data suggest that Dilp8-induced NO inhibition of ecdysteroid synthesis is mediated by the down-regulation of cytochrome P450 enzymes, but it is unclear how NO represses gene transcription. A Lgr3-like GPCR is expressed in the *G. lateralis* and *C. maenas* YO transcriptomes (**Table 2**) (25, 29). An ILP2 is an ortholog of Dilp8 that is primarily expressed in nervous tissue, such as brain and eyestalk ganglia of Eastern spiny lobster (*Sagmariasus verreauxi*); brain and thoracic ganglion of red-claw crayfish (*Cherax quadricarinatus*); and in eyestalk ganglia, brain (males), thoracic ganglion (males), and sperm duct in ornate spiny lobster (*Panulirus ornatus*) (42, 83). It is not known if ILP2 is expressed in limb regenerates, and if there is higher expression in secondary than primary regenerates, as only 2˚ regenerates have LAF_pro_ activity (13). Taken together, these data suggest that LAF_pro_ is an Dilp8-like peptide that binds to Lgr3, activating the MIH signaling pathway to inhibit YO ecdysteroidogenesis (**Figure 3**).

### Ca^2+^/Diacylglycerol/Protein Kinase C Signaling

In the canonical pathway, ligand binding to a GPCR activates phospholipase C (PLC) *via* a G_q_ protein anchored in the cell membrane. PLC converts phosphatidylinositol to diacylglycerol (DAG) and inositol trisphosphate (IP3). DAG and IP3-initiated release of Ca^2+^ from smooth endoplasmic reticulum activate protein kinase C (PKC), which phosphorylates downstream targets to effect metabolic changes. All these components are represented in the KEGG calcium and phosphatidylinositol signaling pathways (**Table 1**) (21). Moreover, the YO has PKC activity (85).

Activation of PKC stimulates ecdysteroid synthesis and secretion by the YO. Studies using pharmacological reagents show that the pathway activating PKC is distinct from the MIH signaling pathway (**Figure 3**). A DAG analog or phorbol 12-myristate 13-acetate (PMA) stimulate PKC activity and ecdysteroid secretion by the *Cancer antennarius* YO *in vitro* (85). PMA counters the inhibitory effects of reagents that stimulate MIH signaling, such as forskolin, dibutyryl cAMP, and cyclic nucleotide phosphodiesterase inhibitor IBMX, and has no effect on cAMP levels (85). By contrast, PMA has the opposite effect on crayfish YO by inhibiting ecdysteroid secretion (86). PLC inhibitor U-73122 has no effect on crab and crayfish YO ecdysteroid production *in vitro*, and there are no changes in IP3 and DAG contents of YOs from intact and eyestalk-ablated crabs (86), suggesting that PKC is not involved in YO activation in early premolt. The downstream targets of PKC are unknown. mTORC1 signaling is likely involved, as PMA stimulates protein synthesis in the YO (84, 85, 87). A potential target of PKC is Akt in the mTOR signaling pathway (**Figure 3**).

The ligand and GPCR for the PKC pathway have not been identified. An intriguing possibility is that PKC is activated by serotonin (5-hydroxytryptamine) and other biogenic amines. The YO expresses serotonin, dopamine, and octopamine GPCRs (**Table 2**) (25, 29). Serotonin, dopamine, and octopamine function as neurotransmitters and neuromodulators in the crustacean central nervous system, but they may also act as neurohormones (46, 60, 88–91). Interestingly, there is evidence that the YO can synthesize serotonin (92). Much of the research of biogenic amines functioning as neurohormones has been focused on their roles in regulating decapod reproduction. For example, serotonin stimulates ovarian maturation, whereas octopamine delays gonadal development and inhibits ovarian maturation (93–95). Serotonin stimulates YO ecdysteroid production *in vitro* in mud crab (*Scylla serrata*) (96). In insects, serotonergic neurons directly innervate the PG and stimulate ecdysteroidogenesis (97–99). Octopamine acts as an autocrine factor that enhances PG ecdysteroidogenesis (100), but its effect on YO ecdysteroidogenesis is unknown. In *C. maenas*, dopamine D2-like and 5-hydroxytryptamine receptor 4 are up-regulated in the YO relative to their levels in the epidermis, although the receptors are not differentially expressed in the YO over the molt cycle (29). In *G. lateralis* YO, two serotonin receptors, designated *Gl-GPCR-A30* and *-A32*, and an octopamine receptor, designated *Gl-GPCR-A34*, show different patterns of expression over the molt cycle (dopamine receptor was not identified in the *G. lateralis* YO transcriptome) (25). *Gl-GPCR-A30* shows higher expression in early premolt and no expression in postmolt animals, while *Gl-GPCR-32* is expressed in all five molt stages with higher expression in postmolt (25). *Gl-GPCR-A34* is expressed in all molt stages, with higher expression during premolt (25). These data suggest that serotonin and octopamine are tropic factors in the YO. However, the signaling pathways activated by biogenic amines differ between insects and crustaceans. In the PG, serotonin and octopamine increase cAMP (97, 98), while in the YO, serotonin and octopamine action may be mediated by Ca^2+^/DAG (**Figure 3**). Future research should be directed to establishing the mode of action of biogenic amines on the YO.

## Receptor Tyrosine Kinases

The receptor tyrosine kinase (RTK) superfamily regulates animal development and homeostasis (101). There are about 58 RTKs in 20 subfamilies in mammals, with fewer in arthropods. *D. melanogaster*, for example, has 20 RTKs distributed among 14 subfamilies (76). RTKs have an extracellular ligand-binding domain, a single-pass transmembrane domain, and an intracellular tyrosine kinase domain (TKD). Most RTKs are heterodimers with each subunit consisting of a single polypeptide. By contrast, the insulin receptor is a heterotetramer, consisting of heterodimers of alpha and beta chains linked by disulfide bonds; the α-chain is completely extracellular and, together with the extracellular domain of the β-chain, binds ligand (101). Ligand binding activates RTK activity; autophosphorylation of the TKD activates MAPK, PI3K/Akt, PLCγ-PKC, JAK/STAT, or Rac-Rho signaling cascades (76, 101). The YO expresses both ILP receptors and growth factor receptors (**Table 3**).

**Table 3:** Tyrosine receptor kinases and ligands expressed in *G. lateralis* MLA or ESA Y-organ transcriptomes (16, 20, 21).

Identity	Contig ID	Transcriptome	ORF (aa)	Partial/Full Length	Top Hit	% Identity
EGF	c202604_g1_i1	ESA	197	Partial	*P. monodon*	60%
FGF	c189642_g1_i1	MLA	202	Full	*L. vannamei*	91%
EGFR	c267955_g1_i3	MLA	1491	Full	*C. opilio*	90%
InsR	contig_69766	MLA	1297	Full	*L. vannamei*	29%
FGFR	c219909_g2_i1	ESA	746	Full	*L. vannamei*	75%

Sequences were identified using a reciprocal BLAST and locating conserved domains with NCBI conserved domain search tool. EGF, epidermal growth factor; EGFR, epidermal growth factor receptor; FGF, fibroblast growth factor; FGFR, fibroblast growth factor receptor; InsR, insulin-like receptor β subunit; ORF, open reading frame (amino acids). Species: C. opilio, Chionoecetes opilio; L. vannamei, Litopenaeus vannamei; and P. monodon, Penaeus monodon.

### Insulin-Like Peptide Receptor Signaling

In insects, ILPs are among the many factors that coordinate organismal growth and organ size and determine the timing of molting and metamorphosis (74, 78, 98, 102–105). A target of ILPs is the PG. Insulin-producing neurons in the brain secrete ILPs, in particular Dilp2, 3 and 5, that stimulate ecdysteroid production by the PG (74, 76, 102, 106). ILP/insulin-like protein receptor (InsR) signaling in the PG is mediated by PI3K/Akt/mTOR (74, 97, 106, 107). The role of ILP/InsR signaling in development and growth of crustaceans is not well understood, but it is likely that it has similar actions. Much of the research on crustacean ILP/InsR signaling has focused on reproduction (108, 109).

Insulin/ILPs are synthesized as a single polypeptide with an N-terminal signal peptide sequence, followed by B, C, and A chains (75, 77, 109). Proteolytic processing removes the signal peptide and excises the C chain, producing a B chain/A chain heterodimer stabilized by inter- and intra-chain disulfide bonds between conserved cysteines (75, 77, 109). In some ILPs, the C chain is not completely removed, producing a polypeptide, in which part of the C chain is retained (77). ILPs have been identified in decapod crustacean transcriptomes (28, 32, 33, 38, 83, 110–114). One of the best characterized ILP is the insulin-like androgenic gland hormone (IAG); it is expressed primarily in the androgenic gland and determines adult male characteristics (42, 83, 108, 109, 115, 116). In *Portunus trituberculatus*, *Pt-IAG* is expressed at very low levels in the YO (116). Other crustacean ILPs are expressed in most tissues, but at differing levels. In the oriental river prawn, *Macrobrachium nipponense*, *Mn-ILP* is expressed in brain, eyestalk ganglia, nerve cord, gonads, hepatopancreas, and muscle in adults (117). *Mn-ILP* expression is highest during the rapid growth stage in younger individuals and during the intermolt stage in older individuals (117). *Sv-ILP1* and *Cq-ILP1* are relaxin-like ILPs that are expressed in brain, antennal gland, gonads, and hepatopancreas (females only) (83, 113).

It is generally accepted that decapod crustaceans, like most invertebrates, express a single functional insulin receptor (InsR) (118). InsR has been biochemically characterized in gill, muscle, and hepatopancreas (119–121). InsR β-chain is expressed in many tissues, including the androgenic gland of male *Macrobrachium rosenbergii* (*Mr-IR*) (122), *Fenneropenaeus chinensis* (*Fc-IAGR*) (123), and *S. verreauxi* (*Sv-TKIR*) (124). Orthologs of *Mr-IR* have been identified in the neuropeptidomes of six other decapod species (33). Interestingly, silencing of *Mr-IR* led to androgenic gland hypertrophy and increased *Mr-IAG* production, but had no effect on somatic growth or sex determination, suggesting that molting and sexual differentiation are not solely dependent on the insulin receptor (122). *S. verreauxi tyrosine kinase insulin receptor* (*Sv-TKIR*), when expressed in a COS-7 cell reporting system, is activated by recombinant Sv-IAG and, to a lesser extent, recombinant human insulin, followed by recombinant Mr-IAG and Cq-IAG (124). In the YO, ESA results in a down-regulation of *G. lateralis-InsR*, which is blocked by rapamycin, suggesting that mTORC1 activity represses *Gl-InsR* expression (16).

**Figure 4:**
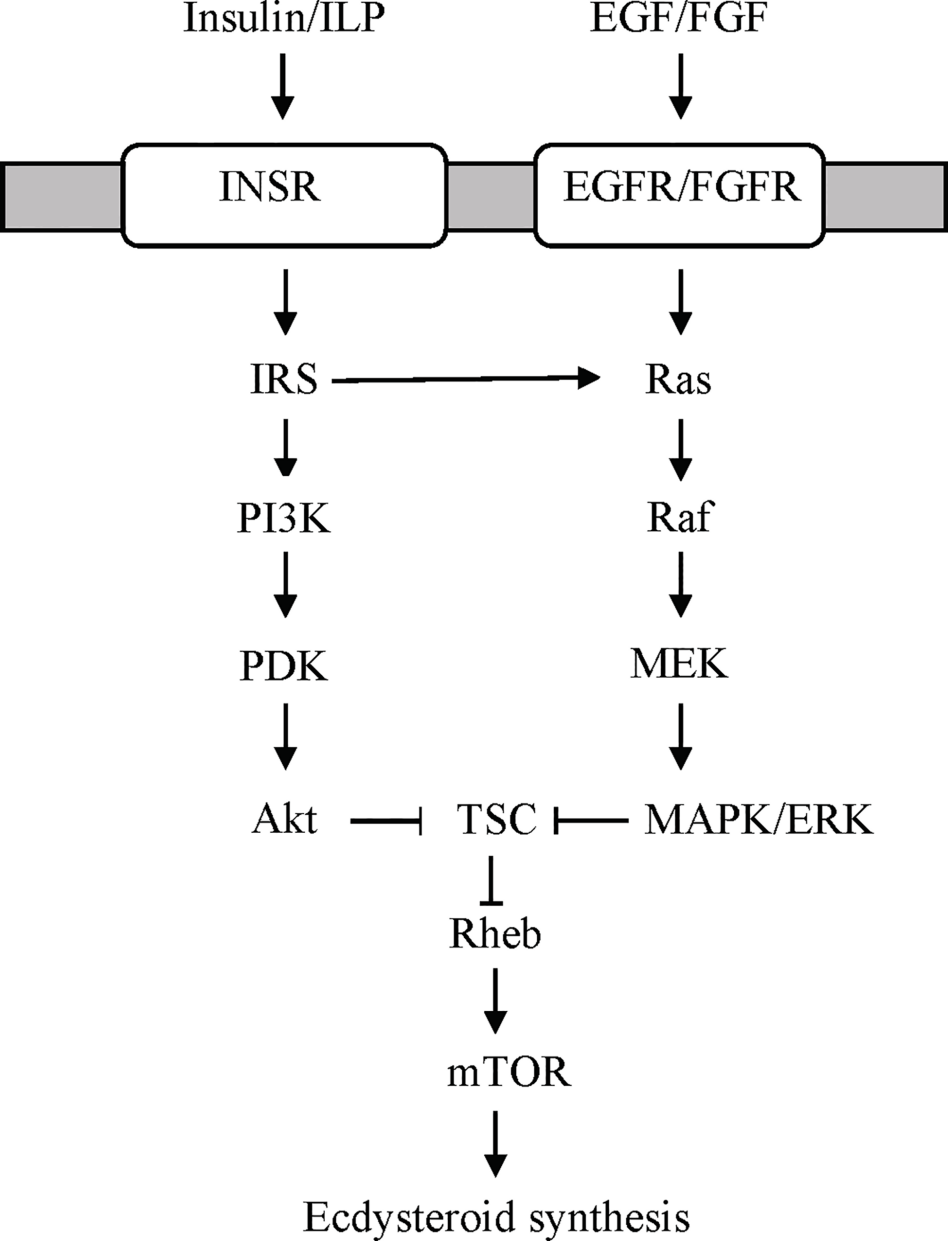
Proposed receptor tyrosine kinase (RTK)-mediated signaling pathways stimulating ecdysteroidogenesis in the YO based on data from the insect prothoracic gland (97, 98). YOs express both types of RTKs (**Table 3**). Growth factors, such as epidermal growth factor (EGF) or fibroblast growth factor (FGF), bind to EGF or FGF receptors, respectively, to activate the Ras/Raf/MEK/ERK signaling pathway. Insulin/insulin-like peptide (ILP) binds to insulin receptor (INSR) to activate PI3K/PDK/Akt and/or Ras/Raf/MEK/ERK signaling. Both pathways converge on mTOR signaling by phosphorylation of tuberous sclerosis complex (TSC) by Akt or ERK, respectively. ERK, extracellular signal-regulated kinase; IRS, insulin receptor substrate; MEK, MAPK/ERK kinase; PDK, protein 3-phosphoinositide-dependent protein kinase; PI3K, phosphoinositide 3-kinase (PI3K).

Remarkably, there are no studies on the effects of insulin or ILPs on YO ecdysteroidogenesis. However, studies of other crustacean tissues indicate that insulin/ILP action is mediated through PI3K/Akt or MAPK/ERK signaling. Bovine insulin increases *Sp-vitellogenin (Sp-Vtg)* mRNA levels in hepatopanceas explants from *Scylla paramamosain* (125). Relatively high concentrations of bovine insulin were needed to elicit a response (>200 ng/ml). The insulin-induced increase in *Sp-Vtg* is blocked by PI3 kinase inhibitor (LY294002) and mTORC1 inhibitor rapamycin (125). Their respective recombinant IAGs increase MAPK/ERK phosphorylation in *M. rosenbergii*, *S. verreauxi*, and *Cherax quadricarinatus* testis explants *in vitro* (124). In insects, ILPs (e.g., Dilps1-6 in *D. melanogaster* and Bombyxin in *Bombyx mori*) stimulate PI3K/Akt signaling and mTORC1-dependent ecdysteroidogenesis in the insect PG (78, 97, 106, 107, 126–129). Based on these data, a model for ILP signaling in the YO is proposed (**Figure 4**). Binding of ILPs, such as ILP2, to InsR activates PI3K/Akt signaling, leading to mTOR activation and increased ecdysteroid synthesis. Alternatively, or perhaps in conjunction with PI3K/Akt signaling, ILP activates MAPK/ERK signaling (**Figure 4**). Both pathways converge on mTOR in animal cells (130, 131).

### Growth Factor Receptor Signaling

Growth factor signaling is mediated by the Ras/Raf/MAPK and PI3K/PDK1/Akt pathways (**Figure 4**) (132). In insects, growth factors, such as epidermal growth factor (EGF), fibroblast growth factor (FGF), platelet-derived growth factor, (PDGF) and vascular endothelial growth factor (VEGF), serve many functions critical for embryogenesis, molting, and metamorphosis (76). Activation of the Ras/Raf/MAPK pathway stimulates ecdysteroidogenesis in the insect PG (97, 106). Although not a growth factor, prothoracicotropic hormone (PTTH) activates this pathway by binding to an RTK encoded by *Torso* (97, 98, 102, 106, 133). PTTH is the primary factor that initiates larval molts in most insects. However, a recent study has shown that EGF receptor (EGFR) signaling supports PG ecdysteroidogenesis during the 3^rd^ larva to pupa transition in *D. melanogaster* (134). The function of growth factors in YO ecdysteroidogenesis is unknown. Growth factor signaling pathways are well represented in the YO. These include the ErbB, MAPK, PI3K/Akt, and Ras KEGG signaling pathways (**Table 1**). Contigs can be assigned to two or more of the KEGG pathways (**Figure 4**) (21). The *G. lateralis* YO transcriptome has contigs encoding 106 MAPK signaling components, 66 of which are differentially expressed over the molt cycle (**Table 1**) (21). Forty-eight contigs assigned to the ErbB pathway, which includes EGFR signaling, have been identified (**Table 1**).

Knowledge of growth factors and their functions in crustaceans is limited. Unfortunately, there are no studies determining the effects of EGF, FGF, or VEGF on YO ecdysteroidogenesis. Immunohistochemical analysis indicated that eyestalk ganglia express a VEGF-like protein and VEGF receptor (135). The broad scale application of transcriptomics has aided the identification of growth factors and their receptors in decapod crustacean tissues. VEGFR is expressed in the embryos of *Macrobrachium olfersi* (136). The VEGF signaling pathway is enriched in hemocytes from pathogen-infected *Eriochier sinensis*, suggesting that VEGF is involved in mounting an immune response (137). *Lv-EGF*, *Lv-EGFR*, and *Lv-VEGFR* are expressed in embryos and larvae of *Litopenaeus vannamei* (138). In *M. rosenbergii*, *Mr-EGFR* is expressed in most tissues, with higher expression in thoracic ganglion, ovary, and testis (139). Knockdown of *Mr-EGFR* slows accumulation of mass in juvenile male *M. rosenbergii*, but has no effect on ecdysis frequency (139). Using transcriptomic data, a cDNA encoding the complete EGFR sequence was cloned from *S. parmamosain* ovary; *Sp-EGFR* is expressed in most tissues, with higher expression in YO, ovary, stomach, heart, and gill (140). Human EGF (1 nM and 10 nM) caused a transient increase in *Sp-Vtg receptor* and *Sp-Cyclin B* mRNA levels in ovary explants; the increases were blocked by pretreatment with EGFR tyrosine kinase inhibitors AG1478 and PD153035 (140). The *G. lateralis* YO expresses *Gl-EGF*, *Gl-FGF*, *Gl-EGFR*, and *Gl-FGFR* (**Table 3**) (16, 20). These data suggest that EGFR functions in a wide variety of tissues, including the YO (**Table 3**). The expression of *Gl-EGF* and *Gl-FGF* suggests that EGF, and perhaps FGF, act as autocrine factors in the YO as EGF does in the PG (134).

## Transforming Factor β/Activin Signaling

TGFβ signaling plays essential roles in animal cell differentiation and homeostasis, and dysregulation of TGFβ signaling contributes to many diseases including cancer (141–143). In the canonical pathway, a ligand binds to TGF receptor 2 (R2) homodimer, which associates with TGFR1 homodimer to form the active heterotetramer receptor complex by TGFR2 autophosphorylation and phosphorylation of TGFR1 and regulatory (R)-Smad (144, 145). Two phospho-R-Smads bind to one Co-Smad and the R-Smad/Co-Smad complex translocates to the nucleus to regulate gene transcription (141, 144, 145). Several proteins inhibit TGFβ signaling. Inhibitory (I)-Smads block TGFβ signaling by either preventing R-Smad phosphorylation by TGFR2 or preventing binding of Co-Smad to phospho-R-Smad. FK-506 binding protein 1A (FKBP12) binds to TGFR1 and prevents phosphorylation of TGFR1 by TGFR2 (141, 146). BMP and activin membrane bound inhibitor (BAMBI) acts as a TGFR1 pseudo receptor, as it binds ligand, but lacks the protein kinase domain for signal transduction. TGFβ signaling cross-talks with many other signaling pathways, such as MAPK, Akt, PKC, CAMKII, GSK3, JAK, JNK, Wnt, Notch, and Hedgehog (145).

TGFβ ligands include bone morphogenic proteins (BMPs), growth and differentiation factors (GDFs), Activin, anti-Müllerian hormone, nodal, and TGFβs (143, 147). They often function as autocrine factors, acting on the same tissue in which they are synthesized and secreted. TGFβ/Activin signaling, in particular, determines the competency of insect and crustacean molting glands to respond to neuropeptides (2, 97, 103). Insects express three Activins: Activin-β (Actβ), Myoglianin (Myo)/Myo-like, and Dawdle (Daw) (148, 149). In *D. melanogaster*, knocking out Activin signaling by targeting R-Smad *dSmad2*, Type I receptor *Baboon* (*Babo*), Type II receptor *Punt*, or Co-Smad *Medea* in the PG leads to 3^rd^ instar arrest and failure of larvae to metamorphose and down-regulation of signaling genes *Torso* and *InR* and Halloween genes *Dib* and *Spok* in the PG (150). In the German cockroach *Blatella germanica*, an increase in *Bg-Myo* mRNA levels in the PG is associated with increased ecdysteroid synthesis at the end of the 5^th^ instar (151). These data indicate that Activin signaling is necessary for the stimulation of ecdysteroidogenesis by PTTH and ILP. Interestingly, knocking out any one of the three Activin ligands Actβ, Myo, or Daw has no effect on *D. melanogaster* molting and metamorphosis (148). All three ligands must be knocked out in the PG in order to manifest the developmental arrest phenotype, suggesting some degree of redundancy between the three ligands and their Babo receptors (148). Adult decapod crustaceans express a single myostatin (Mstn)-like/GDF11 that is related to mammalian Mstn/GDF8 and GDF11 (2, 18). TGFβ/Mstn signaling drives the transition of the YO to the committed state, resulting in the YO becoming less sensitive to MIH and CHH in mid-premolt and late premolt (2, 15, 152). Recently, a cDNA encoding a Dawdle-like factor was characterized in *S. paramansosain*. *Sp-Daw* is expressed primarily in embryos and larvae, suggesting that it plays a role in developmental processes (153). It may also be involved in the innate immune response in adults (153).

*Mstn* is expressed in most crustacean tissues, with generally higher levels in the YO, heart, and muscle (15, 18, 154–161). Most studies have focused on the role of *Mstn* as a negative regulator of muscle growth, which contributes to organismal growth (5). In Chinese shrimp *F. chinensis*, *Fc-Mstn* mRNA levels are inversely correlated with growth traits among individuals from different genetic lineages (162). It appears that Mstn slows muscle growth by inhibiting mTORC1-dependent protein synthesis. In *G. lateralis* claw muscle, increased protein synthesis during premolt is correlated with decreased expression of *Gl-Mstn* and increased expression of *Gl-Rheb*, the activator of mTORC1 (17, 163). Several studies have attempted to knock down *Mstn* expression as a means to promote growth in aquacultural species. Surprisingly, in several cases, *Mstn* ds-RNA injection has just the opposite effect: an increase in molt interval and/or decrease in growth rate in *Penaeus monodon*, *L. vannamei*, and *Fenneropenaeus merguiensis* (155, 159, 164). These studies did not consider off-target effects. Reduced expression of *Mstn* in the YO could have blocked or delayed the progression from early premolt to mid-premolt, thus lengthening the interval between ecdyses. The effects on YO *Mstn* mRNA levels were not examined in these studies. However, injection of *Es-Mstn*-dsRNA or *Es-Activin receptor IIB* (*Es-ActRIIB*) dsRNA into juvenile *E. sinensis* and injection of *Fc-Mstn*-dsRNA into juvenile *F. chinensis* accelerated molting and growth when compared to a control group (154, 165, 166). However, control animals were injected with RNase-free water or phosphate-buffered saline, rather than an unrelated dsRNA construct (154, 164–166). Thus, one cannot rule out a nonspecific response to dsRNA injection. Only two of the studies used dsRNA products of unrelated sequences as controls, and both those showed molt inhibition (155, 159).

Molting alters TGFβ/Activin signaling gene expression in the *G. lateralis* YO. Analysis of RNAseq data of MLA-induced animals shows increases of *Gl-Activin RI*, *Gl-Smad2/3* (*Gl-R-Smad*), and *Gl-Smad4* (*Gl-Co-Smad*) in early premolt to mid-premolt, while TGFβ signaling inhibitors, *Gl-Smad6* (*Gl-I-Smad*) and *Gl-BAMBI*, are down-regulated during premolt (21). By contrast, ESA decreases *Gl-Activin RI*, *Gl-Smad2/3*, *Gl-Smad4*, *Gl-Smad6*, and *Gl-BAMBI* expression (16). The differences between the MLA and ESA transcriptome results are attributed to the ESA study focusing on initial YO activation as the animals did not transition to mid-premolt (16). Neither MLA nor ESA had a significant effect on the relative expression of *Gl-Mstn* in the *G. lateralis* YO transcriptomes (16, 21), although qPCR showed that *Gl-Mstn* mRNA level increases by three days post-ESA (15). Future research should use qPCR to establish the precise timing of the effects of ESA ± SB431542 on the expression of TGFβ/Mstn signaling genes.

## mTOR Signaling

mTOR is a serine/threonine PI3-related protein kinase that allocates energy in response to nutrients, growth factors, and stress in eukaryotic cells at transcriptional and post-translational levels (167–169). mTOR associates with other proteins to form two complexes: mTOR Complex 1 (mTORC1) and Complex 2 (mTORC2) (169). mTORC1 contains Raptor and controls protein translation, lipid and nucleotide synthesis, and autophagy (169, 170). mTORC2 contains Rictor and controls cytoskeletal remodeling, ion transport, and cell survival and proliferation (169, 170). mTORC1 is inhibited by rapamycin, mediated by FKBP12, while mTORC2 is insensitive to rapamycin (167, 169). mTORC1 is activated by GTP/Ras homolog enriched in brain (Rheb). Rheb, in turn, is controlled by the tuberous sclerosis complex (TSC), a heterotrimeric protein composed of Hamartin (TSC1), Tuberin (TSC2), and TBC1 domain family member 7 (TBC1D7) (169). TSC is a GTPase activating protein (GAP) that inactivates GTP/Rheb by promoting the hydrolysis of GTP to GDP (170). Growth factor signaling pathways converge on the TSC to promote cellular growth (**Figure 4**) (170). Phosphorylation of TSC by Akt, ERK, p90 ribosomal S6 kinase 1 (RSK1), and other protein kinases inhibit the GAP activity to prevent inactivation of GTP/Rheb, which leads to mTORC1 activation (169). Phosphorylation of ribosome subunit 6 kinase (S6K) and 4E-binding protein (4E-BP) by mTORC1 increases translation of mRNA to protein.

mTORC1 is required for increased ecdysteroidogenesis in the arthropod molting gland. PTTH stimulates mTORC1-dependent protein synthesis, which increases ecdysteroid synthesis of the insect PG (126, 128, 171–178); see (179) for earlier references). In *D. melanogaster*, *Rheb* overexpression or *TSC2* knockdown in the PG reduces the developmental delay in food-limited 3^rd^-stage larvae, resulting in lower adult weights (180). Additionally, *Rheb* overexpression under food-limiting conditions increases transcription of Halloween genes *Phm* and *Dib* (180). Comparable studies on decapod crustaceans, such as knockdown of *Rheb* and *TSC2* with dsRNA constructs, have not been done. Incubation of hepatopancreas explants with *Sp-Akt*-dsRNA reduced the insulin-induced increase in *Sp-Vtg* mRNA level, but had no effect on *Sp-Rheb* mRNA level (125). Rapamycin inhibits YO ecdysteroid synthesis and secretion *in vivo* and *in vitro* and blocks entry of intermolt animals into premolt and delays the transition from early premolt to mid-premolt in eyestalk-ablated *G. lateralis* (14, 15).

Molting up-regulates many of the mTOR signaling components in the YO. In *G. lateralis*, *Gl-mTOR* and *Gl-Akt* mRNA levels increase in mid-premolt and late premolt stages in MLA animals and *Gl-mTOR*, *Gl-Akt*, and *Gl-S6K* mRNA levels increase by three days post-ESA in eyestalk-ablated animals (14, 15). *Gl-elongation factor 2* (*Gl-EF2*) mRNA level is also increased by MLA and ESA, which is consistent with increased protein synthesis in the YO (14, 15). By contrast, molt stage (intermolt, early premolt, and late premolt) has no effect on *Cm-EF2*, *Cm-mTOR*, *Cm-Rheb*, *Cm-Akt*, and *Cm-S6K* mRNA levels in *C. maenas* (14). SB431542 injection blocks the ESA-induced increases in *Gl-EF2*, *Gl-mTOR*, and *Gl-Akt* mRNA levels and decreases *Gl-Rheb* mRNA level, which suggest that TGFβ/Activin signaling is required for sustained up-regulation of mTOR signaling during premolt (15). RNAseq data expands on the results from qPCR analysis. *Gl-mTOR*, *Gl-Raptor*, *Gl-Rictor*, *Gl-S6K*, and *Gl-Akt* are expressed at high levels during intermolt and early premolt and at their lowest levels during postmolt (21). *Gl-Rheb* expression increases during early premolt and mid-premolt stages (21). ESA increases expression of *Gl-mTOR*, *Gl-Raptor*, *Gl-mLST8*, *Gl-Rheb*, *Gl-Akt*, *Gl-S6*, *Gl-S6K*, *Gl-EIF4E*, and *Gl-EF2* (16). These increases are inhibited by rapamycin, suggesting a positive feedback mechanism in which mTORC1 activity up-regulates expression of mTOR signaling genes (**Figure 1**) (16).

**Figure 5:**
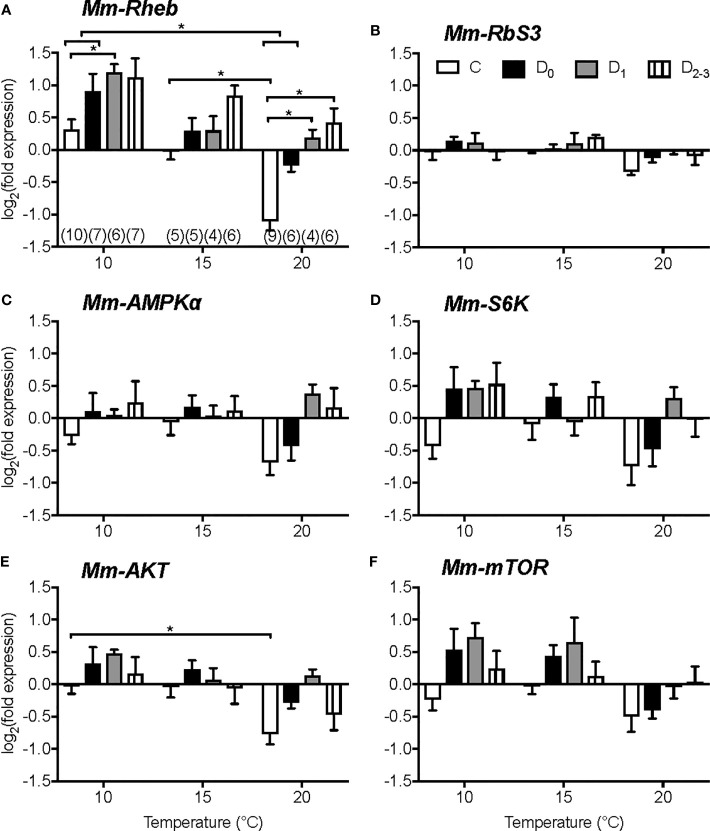
Effects of temperature and molt stage on gene expression in Y-organs of juvenile Dungeness crab, *Metacarcinus magister*. mRNA levels of *Mm*-*Rheb* **(A)**, *Mm*-*ribosome subunit 3* (*RbS3*) **(B)**, *Mm*-*AMPKa* **(C)**, *Mm*-*S6K* **(D)**, *Mm*-*AKT* **(E)**, and *Mm*- *mTOR* **(F)** after 14 days at 10°C, 15°C or 20°C of juveniles in intermolt (C, white), early premolt (D_0_, black), mid-premolt (D_1_, grey), or late premolt (D_2-3_, lines). Data are normalized to the mean absolute mRNA copy numbers in stage C at 15°C. Asterisks denote significant differences at *P* < 0.05. Sample size (*n*) given in brackets below columns in A also apply to the other genes. Data are presented as mean ± 1 S.E.M. *Mm-Rheb* expression is affected by temperature and molt stage. *Mm-Rheb* mRNA level increases during premolt stages and decrease with increasing temperature. From (187).

**Figure 6:**
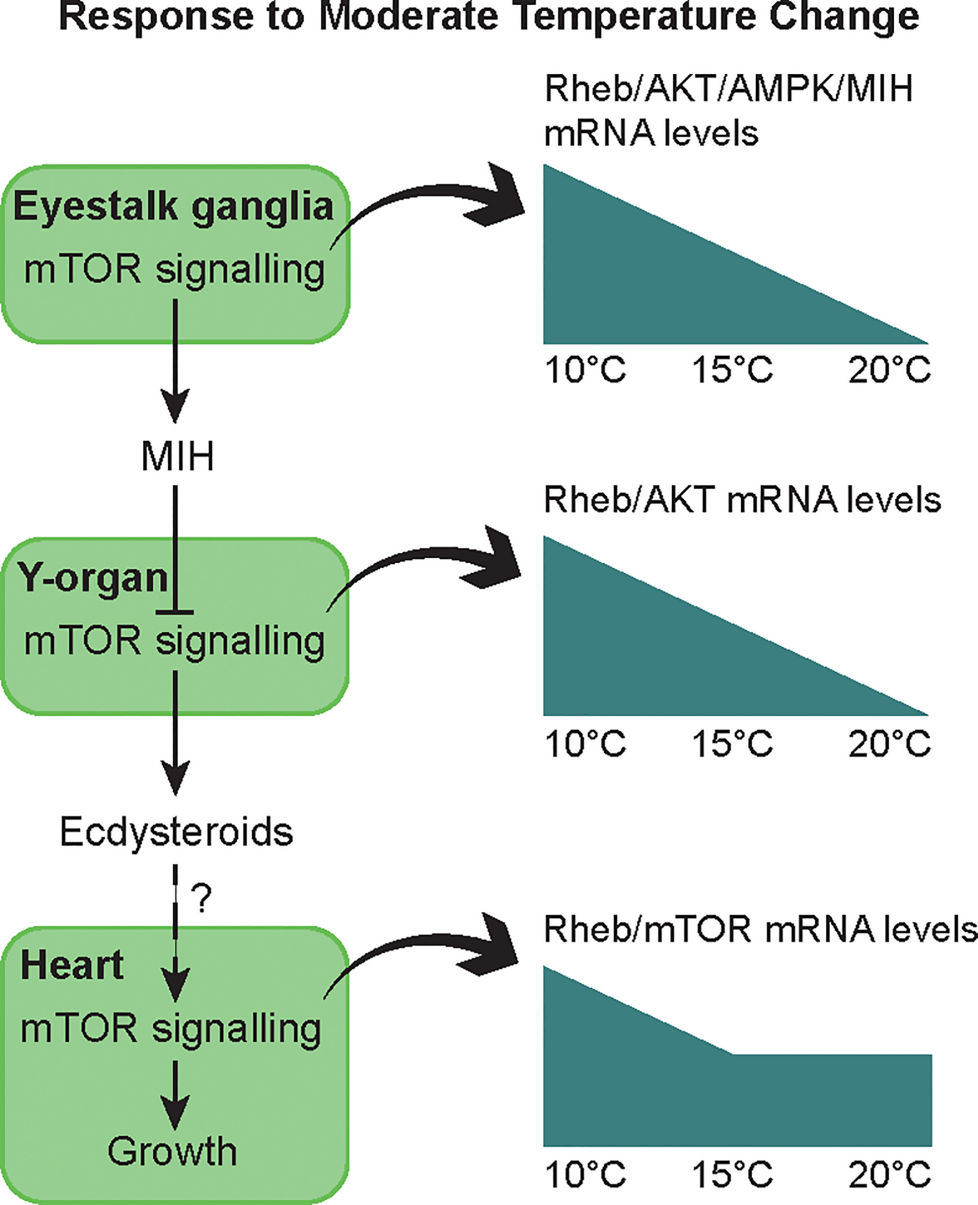
Summary of the effects of moderate thermal stress on molting and growth through the mTOR signaling pathway in juvenile Dungeness crabs. In intermolt, MIH keeps the YO in a basal state with low ecdysteroid secretion by inhibiting mTOR. Low levels of ecdysteroid may stimulate heart muscle growth *via* mTOR signaling. Moderate temperature change (10 to 20°C range) allows acclimation of the animals, at least with respect to some physiological functions. After 14 days, thermal compensation is observed in molt control, i.e. similar ecdysteroid titer across temperatures throughout the molt cycle (187). Mechanisms include up-regulation or down-regulation of *Mm-MIH* and mTOR signaling genes (*Mm-Rheb*, *Mm-Akt*, *Mm-AMPK*) during cold (10°C) or warm (20°C) exposure in the eyestalk ganglia and YO. In the heart, thermal compensation of metabolism is incomplete, as oxygen demand and heart activity increase with temperature. A sustained mRNA level of *Mm-Rheb* and *Mm-mTOR* indicates a greater allocation of energy to maintaining cardiac capacity during warm exposure. From (187).

Temperature affects metabolic processes in crustaceans, including molting, and it is likely that mTORC1 activity contributes to the response of the YO and other tissues to temperature. Within normal physiological ranges, increasing temperature stimulates molting and growth of decapod crustaceans (3, 181–183). However, when an animal reaches its upper thermal limit, molting is inhibited, either directly on the YO or indirectly by inhibitory neuropeptide, such as CHH, secreted by the X-organ/sinus gland (183–186). The effects of temperature on survival, molting, and mTOR signaling gene expression in YO, eyestalk ganglia, and heart were determined in juvenile Dungeness crab, *Metacarcinus magister*. Animals at three different molt stages (12, 19, or 26 days post-ecdysis; these intervals spanned stages C to D_2-3_) were transferred from ambient temperature (~15°C) to 5, 10, 15, 20, 25, or 30°C for 14 days (187). None of the animals transferred to 25 and 30°C survived (187). *M. magister* molt successfully at 21°C, but the growth increment is less than that at 14°C (188); see (187) for additional references). These results indicate that the upper temperature limit for *M. magister* molting success is between 21°C and 25°C. Low temperature (5°C) inhibits molting (187). Between 10°C and 20°C, molt stage progression increases with temperature (187). Gene expression in YO, eyestalk ganglia, and heart is affected by temperature and molt stage, but there is little or no interaction in gene expression between temperature and molt stage (187). In eyestalk ganglia, *Mm-MIH*, *Mm-CHH*, *Mm-Rheb*, *Mm-AMP kinase α subunit (AMPkα)*, and *Mm-Akt* mRNA levels decrease with increasing temperature, particularly at 20°C; *Mm-S6K* mRNA level is not affected by temperature (187). In heart, mRNA levels of *Mm-Rheb*, *Mm-S6K*, *Mm-AMPKα*, *Mm-Akt*, and *Mm-mTOR* are higher at 10°C than at 15°C and 20°C. Of the six genes quantified in the YO, only *Mm-Rheb* expression is affected by both molt stage and temperature (**Figure 5**). *Mm-Rheb* mRNA level is higher in premolt stages (**Figure 5**) and is positively correlated with hemolymph ecdysteroid titers at all three temperatures (187), which suggests that Rheb stimulates mTORC1-dependent ecdysteroid synthesis. *Mm-Rheb* mRNA level decreases with increasing temperature at most molt stages (**Figure 5**). It is noteworthy that only the mRNA level of *Mm-Rheb* is negatively correlated with temperature in all three tissues (**Figure 6**). It appears that the down-regulation of mTOR signaling serves as a compensatory mechanism for higher metabolic rates at higher temperatures, so that energy allocation to protein synthesis is maintained at relatively constant levels (**Figure 6**). Taken together, the data suggest that *Rheb* expression can be used as a proxy to assess the effects of molting and temperature on mTORC1 activity in crustacean tissues.

## Conclusions and Future Research

The control of molting involves the integration of a variety of signals that affects the ecdysteroidogenic capacity and activity of the arthropod molting gland (2, 62, 78, 106). This is reflected by the diversity and actions of the factors involved. In the insect PG, tropic factors, such as PTTH, ILPs (e.g., dILP1-6, Bombyxin), growth factors (e.g., EGF and VEGF), biogenic amines (e.g., serotonin and octopamine), and FXPRLamide peptide, stimulate ecdysteroidogenesis (97, 98, 105). Static factors, such as dILP8, prothoracicostatic peptides, Bommo-myosuppressin, and FMRFamide-related peptide, inhibit ecdysteroidogenesis (97, 98, 105). By contrast, the neuropeptides MIH and CHH are the only known ligands identified for YOs in crustaceans (2, 50, 55, 189, 190). Transcriptomic analysis has revealed that the YO expresses receptors for ILPs, growth factors, biogenic amines, and neuropeptides (**Tables 2**, **3**) (25, 29). The large number of GPCRs in particular indicates that the YO resembles the insect PG in being able to integrate a large number of signals to coordinate organ growth, development, and molting. MIH, CHH, LAF_pro_, and perhaps FMRFamide act as static factors on the YO. Drawing on comparisons with the insect PG, ILPs, EGF, VEGF, corazonin, and LAF_an_ may act as tropic factors. The effects of these and other ligands (e.g., serotonin, octopamine, FGF, dopamine, pigment dispersing factor, allatostatins, ecdysis-triggering hormone, crustacean cardioactive peptide (CCAP), CCHamide, diuretic hormones DH31 and DH44, and Bursicon) on YO ecdysteroidogenesis remain to be determined.

PTTH and MIH are the primary neuropeptides controlling molting in insects and crustaceans, respectively (2, 50, 105, 106). It is remarkable that the control of a process as critical as ecdysis is to organismal growth would have evolved diametrically opposite mechanisms in these two major arthropod groups. PTTH activates the PG, while MIH inhibits the YO. Thus, molting in insects is initiated by the release of PTTH from neurosecretory neurons in the brain, while molting in crustaceans is initiated by reduced MIH release from neurosecretory neurons in the eyestalk X-organ/sinus gland complex (2, 11, 105). In *D. melanogaster*, PTTH stimulates PG ecdysteroidogenesis by binding to the Torso RTK and activating the Ras/Raf/MAPK signaling pathway (106). In lepidopterans (*Manduca sexta* and *Bombyx mori*), a PTTH-induced Ca^2+^ influx activates both Ras/Raf/MAPK signaling and cAMP-dependent signaling and both contribute to a large increase in ecdysteroid synthesis (97, 105, 179). MIH inhibits YO ecdysteroidogenesis by binding to a putative GPCR and activating a cyclic nucleotide-dependent signaling pathway (2, 50). A cAMP/Ca^2+^-dependent triggering phase is linked to a NO/cGMP-dependent summation phase to inhibit the YO between MIH pulses (2, 50, 55). RTKs in the YO most likely function to stimulate ecdysteroidogenesis, as they do in the PG (76, 97, 98).

**Figure 7:**
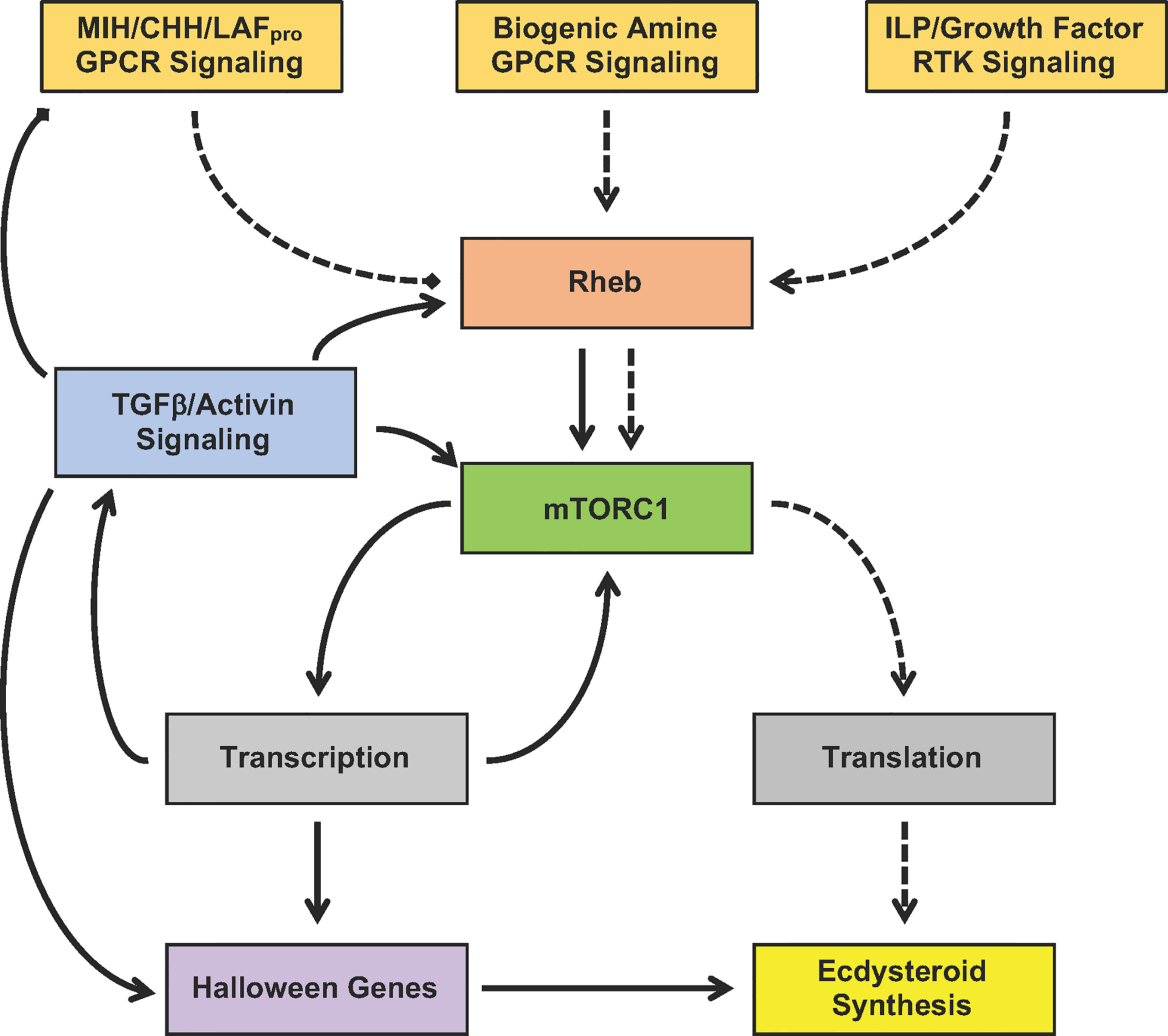
Proposed model for the signaling pathways mediating YO activation (dashed lines) and commitment (solid lines). Signaling pathways converge on mTORC1 by controlling Rheb activity. MIH/CHH/LAF_pro_ GPCR signaling inhibits mTORC1 by inactivating Rheb, while biogenic amine GPCR signaling and ILP/growth factor RTK signaling stimulates mTORC1 by activating Rheb. YO activation during early premolt requires mTORC1-dependent global translation of mRNA to protein, which leads to increased ecdysteroid synthesis. YO commitment involves mTORC1-dependent changes in gene transcription, resulting in up-regulation of TGFβ/Activin, Rheb/mTORC1, and Halloween genes and down-regulation of MIH/CHH/LAF_pro_ GPCR signaling genes.

The YO undergoes phenotypic changes over the molt cycle. The molt cycle is unidirectional, with YO transitions occurring at critical checkpoints that determine progression to the next molt stage. **Figure 7** presents a working model for the signaling pathways that control YO basal (intermolt stage), activated (early premolt stage), and committed (mid- and late premolt stages) phenotypes. The most important decision is to initiate molting, which is determined by the integration of external cues, most likely mediated by the brain and eyestalk ganglia, which control the release of MIH from the X-organ/sinus gland complex, and internal cues, such as nutritional status, organ size, and limb regeneration, that act directly on the YO (2, 3, 11). The signaling pathways converge on Rheb/mTORC1 to regulate ecdysteroidogenesis (**Figure 7**). Activation of GPCR signaling by static factors (e.g., MIH and CHH) inhibits Rheb/mTORC1, maintaining the YO in the basal state. Activation of RTK signaling by tropic factors (e.g., ILPs and growth factors) stimulates Rheb/mTORC1. Although RTK signaling can potentially activate the YO, MIH release prevents molt initiation until environmental conditions are met. YO activation is mediated post-translationally by mTORC1-dependent increased global translation of mRNA to protein, resulting in increased ecdysteroid synthesis and secretion (**Figure 7**, dashed lines). The rising ecdysteroid titers in the hemolymph mark the entry into early premolt (1). The activated YO remains sensitive to static factors, as ecdysteroidogenesis is inhibited by MIH, CHH, and LAF_pro_ (2). During early premolt, the YO synthesizes and secretes Mstn-like factor, which binds to Activin receptors to activate Smad transcription factors, leading to down-regulation of MIH signaling genes and up-regulation of Rheb/mTORC1 and Halloween genes in the committed YO (**Figure 7**, solid lines).

Although much progress has been made over the last ten years, many questions remain and research efforts should be directed at answering them:

1. What is the identity of the MIH receptor? The evidence indicates that the MIH receptor is a GPCR and several potential candidates have been identified from *in silico* analysis of YO transcriptomes (**Figure 2**) (25). Moreover, there is evidence from studies of lobster muscle that the CHH receptor is a membrane receptor guanylyl cyclase (GC-II) (8, 56, 179, 191). A heterologous reporting system in COS-7 cells holds promise as a functional assay for quantifying the specificities of candidate GPCRs and GC-II to recombinant MIH and CHH (124).
2. How does MIH signaling inhibit mTORC1? The current thinking is that MIH inhibition of ecdysteroidogenesis is mediated by PKG (2, 56). The downstream substrates of PKG are unknown. A possible target is TSC (**Figure 3**), in which phosphorylation stimulates GAP activity, although it is not known if TSC is phosphorylated by PKG (192). Proteomic analysis using liquid chromatography-tandem mass spectrometry now provides the technology to identify and quantify phosphoproteins in the YO in response to rMIH and PKG inhibitors. A similar approach was used to show that NO synthase is phosphorylated in the activated YO (7).
3. What is the identity of LAF_pro_? LAF_pro_ is a peptide factor produced by secondary limb buds to delay molting (13). As discussed in Section 2.3, the discovery of an Lgr3 GPCR in the YO transcriptome suggests that LAF_pro_ is a Dilp8-like peptide that inhibits ecdysteroidogenesis *via* the MIH signaling pathway (**Figure 3**). Like the YO, inhibition of PG ecdysteroid synthesis by Dilp8 is through the activation of NO synthase (73, 79). This suggests that the inhibition of ecdysteroidogenesis by NO/cGMP/PKG is conserved in arthropod molting glands.
4. How does mTORC1 control gene expression? mTORC1 activity alters the mRNA levels of thousands of genes, including those for mTOR, TGFβ, and MIH signaling and ecdysteroidogenesis (16), presumably by altering the activities of transcription factors and co-factors. In *D. melanogaster*, the transcription factors Krüppel homolog 1 (Kr-h1), seance, ouija board, molting defective, ventral veins lacking, and Knirps are linked to Halloween gene expression (193–195). *Kr-h1* is a critical component of the juvenile hormone (JH)/methyl farnesoate (MF) signaling pathway in insects (196–198), and recent studies indicate that *Kr-h1* plays a role in crustacean development and reproduction (199–203). The YO expresses *Kr-h1* and other MF signaling components, suggesting that it also has a role in molt regulation (204). One potential function is in the down-regulation of Halloween gene expression when the YO transitions to the repressed state in late premolt.
5. What are the gene targets of TGFβ/Activin signaling? TGFβ/Activin drives the transition of the YO from the activated to the committed state. It is hypothesized that Smad transcription factors, activated by Mstn-like factor, up-regulate the expression of commitment genes that determine the phenotypic properties of the committed YO (**Figure 1**), such as low sensitivity to MIH, CHH, and LAF_pro_ and high ecdysteroid production. Possible targets are Rheb/mTORC1, MIH signaling genes, and Halloween genes (**Figure 7**). These and other gene targets can be identified by determining the effects of ESA ± SB431542 on the YO transcriptome and proteome.
6. What are the mechanisms mediating the transitions of the YO from the committed to repressed state and from the repressed to basal state? The repressed YO is transcriptionally inactive and has very low ecdysteroid synthesis, leading to low hemolymph ecdysteroid titers during the postmolt stage. It is hypothesized that the ecdysteroid peak at the end of premolt triggers the repressed state, mediated by ecdysteroid receptor (EcR/RXR) and ecdysone-response proteins. Even less is known about what causes the YO to return to basal state at the end of the postmolt stage. Perhaps a signal from the integument, upon completion of exoskeleton synthesis marked by the deposition of the membranous layer, is involved.
7. What is the role of RTKs in regulating ecdysteroidogenesis? Our understanding of RTKs and their ligands in the YO is largely based on inferences from research on the insect PG. It is hypothesized that ILPs and growth factors stimulate ecdysteroidogenesis (**Figure 4**), but their effects are dampened or nullified by MIH. The YO expresses EGF and FGF, suggesting that both have an autocrine function. *In vitro* assays can determine the effects of recombinant insulin, EGF, and FGF on YO ecdysteroid synthesis and secretion.
8. Is ecdysteroidogenesis regulated by biogenic amines and neuropeptides other than MIH and CHH? The YO expresses a large number and diversity of GPRCs. Of the 99 GPCRs in the *G. lateralis* YO, 65 are assigned to known receptors (25). Of particular interest are GPCRs for corazonin, serotonin, and octopamine, which stimulate ecdysteroidogenesis (**Figure 3**; Sections *Corazonin Receptor* and *Ca^2+^/Diacylglycerol/Protein Kinase C Signaling*). However, other ligands involved in molting, such as ecdysis triggering hormone and Bursicon, should also be investigated.
9. What are the roles of Wnt, Hedgehog, Notch, and Hippo signaling pathways in the YO? These pathways are implicated in controlling ecdysteroidogenesis in the insect PG (74, 97, 98, 205) and are well represented in the YO transcriptome (**Table 1**). Decapods express a large number of Wnt ligands (206). *Wnt4* is implicated in having roles in limb regeneration and the immune response in decapods (207, 208). *Gl-Wnt5* and *Gl-Wnt7* are expressed at their highest levels in late premolt, suggesting that these ligands are involved with the ecdysteroid peak and transition of the YO to the repressed state (21). This constitutes an entirely new area of research in the coming years.

Transcriptomics and proteomics have revealed the complexities of the regulation of the arthropod molting gland. These approaches have been successfully applied to insect PG and complement functional genetic studies on *D. melanogaster* (176, 205, 209–215). Transcriptomic and proteomic analysis of the YO has revealed that the PG and YO are more similar than they are different. The PG and YO express the same KEGG signaling pathways. Many of these signaling pathways converge on mTORC1, which plays a central role in regulating ecdysteroid synthesis in both endocrine organs. A second shared property is that TGFβ/Activin signaling alters the ligand sensitivity of the molting gland. In insects, Activins increase the sensitivity of the PG to PTTH in preparation for the large ecdysteroid peak prior to the metamorphic molt, whereas Mstn/Activin decreases the sensitivity of the YO to MIH and CHH in mid- and late premolt. The great diversity in GPCRs indicates that the YO, like the PG, can respond to a variety of ligands, some of which are inhibitory and others are stimulatory. As RTKs stimulate ecdysteroidogenesis in the PG, it seems reasonable to postulate that RTKs have the same function in the YO. The study of insect molting and metamorphosis has informed research on crustaceans, but this does not mean that one can fully understand the control of molting in crustaceans by studying *Drosophila*. There are fundamental differences in evolutionary history and life history between insects and decapod crustaceans and even between insect orders. The lineages that gave rise to insects and crustaceans have been separated for more than 500 million years, allowing time for the evolution of divergent ligands and signaling pathways to become dominant (216, 217). Unlike insects, most decapod species continue to molt as adults, enabling them to grow to larger sizes. The larger size is a distinct advantage, as one can obtain the amount of YO tissue needed for transcriptomics from two or three individuals (16, 20, 21, 29) and proteomics (24, 218). This allows for increased sample sizes for statistical analysis and potentially more experimental treatments and time points for the study of molting gland function. Thus, crustacean models complement insect models for achieving a broader understanding of how arthropods integrate growth control with external and internal cues.

## Author Contributions

The author confirms being the sole contributor of this work and has approved it for publication.

## Funding

This research was supported by grants from the National Science Foundation (IOS-1257732 and IOS-1922701).

## Conflict of Interest

The author declares that the research was conducted in the absence of any commercial or financial relationships that could be construed as a potential conflict of interest.
